# Improving working equine welfare in ‘hard-win’ situations, where gains are difficult, expensive or marginal

**DOI:** 10.1371/journal.pone.0191950

**Published:** 2018-02-06

**Authors:** Joy Pritchard, Melissa Upjohn, Tamsin Hirson

**Affiliations:** Brooke, London, United Kingdom; Universidade do Porto Instituto de Biologia Molecular e Celular, PORTUGAL

## Abstract

**Purpose:**

Brooke is a non-government organisation with working equine welfare programmes across Africa, Asia and Latin America. In 2014, staff from ten country programmes were asked to identify ‘no-win’ situations (subsequently reframed as ‘hard-wins’)—where improving equine welfare is proving difficult, expensive and/or marginal—in order to inform strategic decisions on how to approach, manage and mitigate for such situations.

**Methods:**

The Delphi-type consultation process had three phases. Round 1 posed five questions in the form of a workshop, survey and semi-structured interviews. Round 2 re-presented key themes and sense-checked initial conclusions. Round 3 reviewed the nature and prevalence of hard-win situations at an international meeting of all participants.

**Results:**

Reasons given for hard-win situations included: no economic or social benefit from caring for working animals; poor resource availability; lack of empathy for working equids or their owners among wider stakeholders; deep-seated social issues, such as addiction or illegal working; areas with a high animal turnover or migratory human population; lack of community cooperation or cohesion; unsafe areas where welfare interventions cannot be adequately supported. Participants estimated the prevalence of hard-win situations as 40–70% of their work. They suggested some current ways of working that may be contributing to the problem, and opportunities to tackle hard-wins more effectively.

**Conclusion and animal welfare implications:**

Respondents agreed that if equine welfare improvements are to span generations of animals, interventions cannot rely on relatively simple, technical knowledge-transfer strategies and quick-wins alone. Programmes need to be more flexible and iterative and less risk-averse in their approaches to embedding good equine welfare practices in all relevant actors. Consultation recommendations informed development of Brooke’s new global strategy, a revised organisational structure and redefinition of roles and responsibilities to streamline ways to approach hard-wins in the complex environments and socio-economic contexts in which working equids are found.

## Introduction

There are an estimated 100 million working equids worldwide, supporting hundreds of millions of households and small businesses [1–2]. Brooke is an equine welfare non-government organisation (NGO) working in ten countries in Africa, Asia and Latin America to improve the lives of working horses, donkeys and mules. In 2014, Brooke held a strategic review of scenarios where the owners or users of working animals are not motivated to improve equine welfare, because better welfare does not improve their economic status.

Put simply, the rationale presented to animal owners is that better husbandry and empathy will improve welfare, which will in turn improve working ability, for example fewer days when the animal is sick, lame or cannot work at all. Economic and wider livelihood benefit is seen as a key trigger to engage community stakeholders in equine welfare-related activities and motivate them to change harmful behaviour towards animals [3]. Linking welfare to livelihoods is also thought to be vital when attempting to influence policy-makers and other key stakeholders which rarely recognise the value of working equids at either household and national levels.

**Fig 1:**
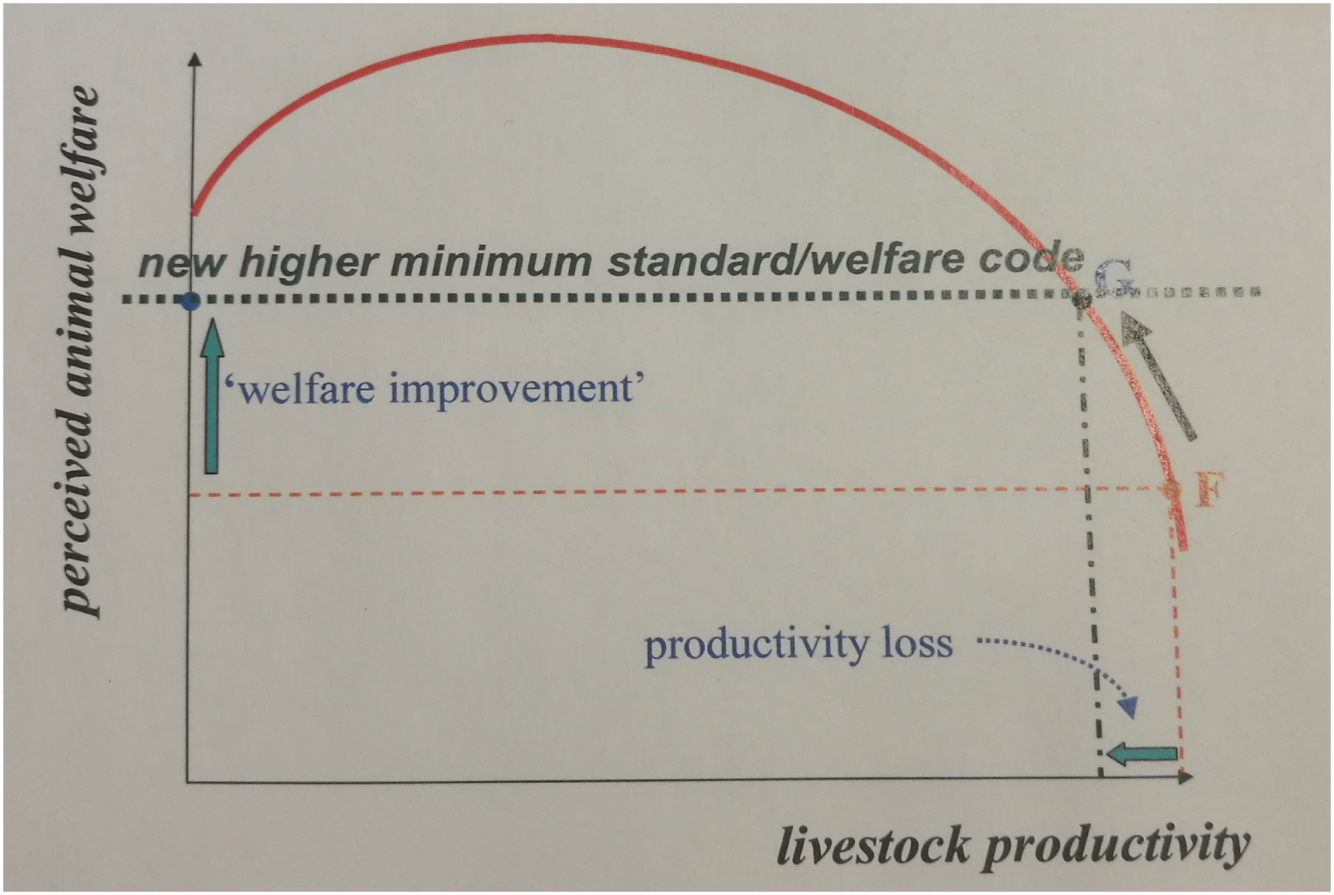
Livestock productivity versus welfare model. Commercial choice between animal welfare and productivity, adapted from McInerney [4]. If poor health, working conditions and/or environment place a working animal between G and F on the graph, the only way to improve welfare is to reduce productivity, leading to a trade-off between animal and human welfare.

Situations where there appears to be no clear link between improved equine welfare and simultaneous improved economics are challenging. Such animals are represented on the far right hand side of the conceptual model of livestock productivity versus welfare described by McInerny [4] Fig 1. An acute dilemma or trade-off between animal and human welfare may result. These could be described as ‘no-win situations’ for the working partnership between animal and owner.

The purpose of the review was to identify and analyse situations where making a long-term, sustainable difference to working equine welfare has been unsuccessful, despite extensive experience and application of resources. These include direct provision of services, capacity building activities for owners and local service providers, and facilitation of access to key welfare resources such as water, foodstuffs and raw materials for essential equipment and infrastructure. Delphi-type consultation techniques, which have been widely used in social policy-making, including veterinary and equine welfare scenarios [5–6] were applied using a variety of communication tools to enable people working in diverse international locations. to feed into the conversation and comment on the views of other participants. Responses reflected experiences gained across South Asia, Africa and Central America and generated from working with owners and their equids operating in industrial, urban, peri-urban and rural agricultural contexts.

The Canadian NGO Engineers without Borders is acknowledged as leading in recognising, analysing and publicly sharing its stories of programmatic struggle and failure, in the name of transparency, accountability and most importantly to foster creativity and learning [7]. An equivalent trail-blazer has not yet emerged in the animal welfare sector. This paper describes a retrospective analysis of real world, long-term animal welfare interventions in a complex international context, in order to learn from difficulties and failures as well as easy-wins and successes.

## Methods

A Delphi-type consultation was carried out between March and May 2014. It involved three rounds of contact with participants; each round shared the findings of the previous one and invited further discussion and comment. The method of presentation varied within and between rounds according to the practicality of gathering participants for face-to-face discussions and the availability of alternatives, such as internet access. Most respondents collected information from a wider group of key informant staff such as veterinary, community engagement staff and programme managers before responding.

Emerging themes from each round were documented by TH and re-presented to respondents in subsequent rounds to ensure that all respondents agreed with the consultation outcomes. TH was present at all meetings to ensure clarity of understanding and accurate record-keeping.

### Round 1

A description of the consultation purpose and process was emailed to Brooke’s Lead Representatives in Afghanistan, Ethiopia, Guatemala, India, Jordan, Kenya, Nicaragua, Nepal, Pakistan and Senegal. A questionnaire posed two contextual questions, described as ‘no-win situations’ and flagged as a major organisational challenge: What happens if improving welfare will not bring about an economic benefit for the stakeholders involved? Will they still be engaged and responsive to welfare improvement activities? This was followed by five specific questions, S1 Table. The country lead representatives were invited to participate on behalf of their programme, with the option to consult wider within their own team where appropriate. Country lead representatives consented verbally to participate in the consultation as part of routine organisational management discussions. At the time of the consultation, the country lead representatives’ length of service in the working equine sector ranged from one year to ten years.

Participants were encouraged not to feel limited by the contextual or specific questions and to add any other information that they thought relevant. International respondents were contacted via an email survey or individual semi-structured interviews. UK respondents were consulted using semi-structured interviews for those directly involved with equine welfare, and a two-hour group workshop for a cross-section of programmatic, fund-raising and communications staff, to gain a non-welfare-specialist perspective.

### Round 2

Following the first round of consultation, TH combined email responses and her own meeting records and extracted their main themes. Emerging themes were re-presented to respondents to ensure that this accurately represented their understanding of initial contributions

The summary was emailed to international staff and presented for discussion in a short workshop to the same UK staff as in Round 1.

### Round 3

Senior staff from all participating national programmes took part in a two-hour face-to-face discussion at an international meeting. They were asked to provide case studies describing where their programme had struggled or not succeeded in bringing about equine welfare improvements, including: the nature of the welfare problem(s); interventions tried; interventions considered but not tried; duration of effort; reasons and root causes for the struggle or lack of success; final outcome; and anything that they would do differently with the benefit of hindsight. They were asked which situations were the most intractable in their country programme i.e. their ‘hard-wins’, and to estimate the proportion of their workload consisting of animals in these situations.

## Results

Eight national programmes responded to Round 1. Participants suggested that describing scenarios as ‘win’ versus ‘no-win’ was too simplistic. They described improving working equine welfare through animal health and community engagement interventions as a continuum, from easier wins to challenging situations that take longer and require more investment of time, effort and resources. As a result, ‘no-win’ situations were reframed for subsequent rounds as ‘hard-wins’, defined as situations where welfare improvement is difficult, expensive and marginal. The exception to this was insecure (mainly conflict-affected) areas, where safe access to working animals and their owners was impossible: these were seen as true ‘no-wins’.

Lack (or perceived lack) of economic benefit was acknowledged as a barrier to equine welfare improvement. However, respondents described barriers that were equally or more important, such as lack of resource availability or opportunity. Economic and non-economic reasons for poor welfare are shown in Table 1.

**Table 1:** Hard-win situations for improving equine welfare described in Round 1 of the consultation. Non-economic situations were described by participants as ‘External’ (outside the organisation’s immediate sphere of influence) and ‘Internal’ (relating to the organisation’s current ways of working).

ECONOMIC HARD-WIN SITUATIONS
Prioritising earnings over welfare improvement, especially where earning capacity is limited (e.g. one market day per week, seasonal work or limited access to land for cultivation)
Inability to afford resources and services such as feed, shelter, improved farriery and cart repairs
Situations where buying a new animal was more economically viable than treating illness or improving the welfare of the existing one
NON-ECONOMIC HARD-WIN SITUATIONS
EXTERNAL:
Difficulty in accessing animals and their owners over the duration of welfare intervention, due to migratory work patterns and/or high animal turnover
Difficulty in identifying the causes of poor welfare
Identifying too many causes of poor welfare, or too much complexity, to design practical interventions
Lack of availability of resources and services, including very basic welfare needs such as water or euthanasia services in Ethiopia
Overwhelming or intervention-resistant environmental issues, such as massive tick infestations in Guatemala
Underlying social issues including drug, alcohol and solvent addiction, domestic violence and illegal working
Presence of traditional or cultural myths and practices that are harmful to equine welfare
Lack of institutional support for working equine welfare among research, veterinary, legislative and other structures and systems.
INTERNAL:
Lack of clarity on the concept of animal welfare
Lack of clarity or pragmatism in the messages and methods used to engage animal owners
Previous interventions influencing animal owners’ expectations or level of participation in current activities
Difficulty in capturing intervention impact (identifying welfare improvement) using currently available welfare assessment tools and timescales

In Round 2, all respondents agreed with the shared summary of Round 1 responses and themes. Respondents recognised that some hard-wins resulted from a mixture of (or interaction between) internal and external factors (see Table 1). They subsequently thought about hard-win situations for working equine welfare as resulting from lack of motivation, lack of opportunity or lack of capability (knowledge), as used in the human health sector [8]. This classification involved degrees of overlap and complexity; for example, improving farriery was mentioned as a notable hard-win in India.

Table 2 summarises the main themes arising from the first two rounds of consultation, with examples of hard-wins, reasons and deeper root causes. In many cases, similar reasons were given by more than one national programme but in contrast, respondents sometimes gave different explanations for the same problem. Some national programmes work with and through local partner organisations. Their additional constraints were described in terms of dilution of welfare knowledge as it is passed on, and also important differences in mission or strategic goals between partner NGOs and Brooke, such as whether animals or humans are the ultimate intended beneficiaries.

**Table 2:** Hard-win themes and examples arising from Rounds 1 and 2, with suggested reasons or root causes.

Major themes	Examples^1^	Reasons/ Root causes
Areas with a high turnover of animals and/ or fragmented or migratory human populations	Donkeys, mules and horses and their owners working seasonally in brick kilns (India, Nepal)	Supporting organisation cannot work consistently with communities, limiting the effectiveness of community engagement interventions
	Sick or injured animals bought cheaply and either worked until no longer fit (Nepal) or improved and sold on for profit (India)	Animal health providers can not follow up individual cases, limiting the effectiveness of healthcare interventions
		Lack of peer support among animal owners to make or sustain welfare changes
Animals are rented/ hired but not owned	Donkeys rented for day-labour pulling goods carts (Ethiopia, Kenya)	No economic benefit for users to improve everyday welfare. Their priority is to earn as much as possible; tomorrow or next season they may have a different animal.
	Tour guides renting animals to carry seasonal tourists to remote Himalayan pilgrimage sites (India)	
Lack of community cohesion or cooperation, even when benefits are mutual	Communities in close proximity but lacking cohesion and sometimes in conflict (Jordan)	Limits the opportunity to use some current approaches such as forming equine welfare groups which enable owners to collaborate to overcome problems.
	Urban and peri-urban environments (Nicaragua, Kenya)	Lack of organisational clarity about what motivates behaviour change in urban contexts, so interventions may not be appropriate or effective
External barriers to welfare improvement despite motivated owners/ users	Working conditions in brick kilns are dictated by the brick factory owner, e.g. no watering points, high brick-making quotas which encourage owners to overload animals (India, Nepal)	Although animal owners are the key actors to improve welfare, they do not have the decision-making power to make the changes needed
Animal-owning communities with deep-seated social issues such as drug, alcohol or solvent abuse, or who are using equids to work illegally.	Workers in illegal sand mines who have drug addiction problems (India)	People are unwilling to meet if working illegally. Supporting organisation does not have the specialist expertise to work with these issues. Equine welfare improvement activities are affected by interruptions and poor attendance.
	Youths with solvent abuse problems renting donkey carts to run small businesses (Kenya)	
No financial value in equine care because replacing the animal is more financially viable than treatment or other welfare improvement measures	Animals are relatively inexpensive compared to resources and services for welfare improvement (Senegal)	No economic motivation to improve welfare
	Owners are wealthy enough to replace animals easily (horse owners in Jordan)	
	Animals are bought very cheaply because they are diseased or injured, to be used until they are no longer capable of working (brick kilns in India, Nepal)	
Lack of resources to improve equine welfare	Absolute lack of resources, such as food and water (parts of Ethiopia)	Although animal owners are the key actors to improve welfare, they do not have the resources to make changes
		Circumstances force owners to prioritise short-term human needs over equine welfare, even if improved welfare would bring long-term benefits to people as well as animals
Lack of services to improve equine welfare	Lack of quality farriery services (India)	Farriery tools are not available, good farriery takes time and costs more
	Absence of equine healthcare infrastructure (Guatemala)	Veterinary services are not available, particularly in remote rural communities. More complex veterinary services for severe disease or injury are only available to wealthy owners in large urban centres, if at all
Inability to euthanase equids in extreme suffering, leading to abandonment and prolonged painful deaths	Epizootic lymphangitis cases (Ethiopia)	Local (often cultural) attitudes towards euthanasia prohibit owners from giving consent
		Appropriate methods and trained personnel are not available
		No economic benefit for an owner or a private service provider to euthanase an animal: owner has to pay for drugs that will not cure the animal, service provider loses income from attempted treatment
Lack of empathy for animals/ working equids and/or their owners among animal health workers or in wider society	Animal health providers (including veterinarians, paraprofessionals and community animal health workers) with little empathy or interest in welfare, who only treat equids to make a profit	Irresponsible use of veterinary drugs (including inappropriate treatment and over-treatment)
		Not interested in treating equids as make more profit from other species
		Not interested in providing a quality, reliable service
		Not interested in providing a service outside limited working hours
Traditional/ cultural practices harmful to equine welfare	Ear-notching, nose-slitting, branding (Kenya)	Traditional for humans as well in some donkey-owning communities (e.g. Maasai) so not seen as a problem
Owners who do not want to improve welfare because this brings unacceptable consequences	Owners of entire male donkeys (Qalander communities in India)	Animals in good welfare are harder to manage and handle
		Animals in good welfare are a target for theft
		Castration is not available or is culturally unacceptable

^1^ Countries in brackets reflect responses of country programme staff to this consultation. The same or similar issues are known to be present in other countries.

Table 3 lists the most challenging hard-win situations and estimated scale of hard-wins in each country, as discussed in the Round 3 international workshop. One respondent highlighted that the scale of hard-win situations may increase over time, as a proportion of a programme’s overall workload, as the easy-wins are achieved and the hard-wins remain unsolved. Overall, the range and scale of hard-wins was considerably higher than expected at the outset of the consultation.

**Table 3:** Worst hard-wins and estimated scale of hard-win situations in each country programme.

Country programme	Worst hard-win situation(s)	Estimated scale of hard-wins (% of total programme workload)
Afghanistan	Lack of water	45%
	Migratory communities	
East Africa office^1^	Lack of water and feed (arid and semi-arid areas)	45%
	Hired animals (high potential areas)	
Ethiopia	Overloading animals in urban areas	55%
	Lack of water	
Guatemala	Security/ staff safety	50%
	Absolute lack of money in poorest 10% of owners	
India	Migratory communities	55–60%
	Poor farriery	
Jordan	Fragmented communities (donkey-owners)	70%
	Tourism economy—emphasis on short-term gains	
Nepal	Brick kilns	40%
Nicaragua	Urban areas	70%
Pakistan	Fragmented communities	30%
West Africa office^2^	Cart drivers (hired animals)	40%
	Migratory communities	

^1^ Representing Kenya

^2^ Representing Senegal

Participants suggested the following changes or improvements to current ways of working:

1. More investment in understanding people’s behaviour towards working animals, so programmes are designed to resolve underlying causes of poor welfare rather than symptoms. Current assumptions about what drives the behaviour of animal owners, users and other actors may be incorrect.
2. Fewer didactic advisory services or teaching/ training events and more facilitation of owners to find their own solutions or mitigation for hard-win situations. This requires further investment in recruiting skilled and experienced facilitation staff from other development sectors and bringing those skills together with animal welfare perspectives and needs.
3. Improving empathy and appreciation for animals (and especially working equine animals) in wider society, for example by increasing public campaigning and advocacy and by targeting younger generations.
4. Reduction in NGO provision of free or subsidised resources and services, which may be contributing to a hand-out culture (although not all participants agreed with this). Brooke’s organisational rationale for reducing its free or subsidised services is that whilst free veterinary treatment engages owners rapidly in the short term, it may discourage owners from making long-term changes to their husbandry and work practices. It may also undermine or displace more sustainable, local animal health services and create a level of expectation among animal owners that cannot be met by the private sector at a later stage.
5. Recognise and accept when interventions are not being effective. Consider withdrawing if all other options have been exhausted. Some situations, such as poor security or very socially complex issues, may be too challenging for a single NGO to address within its charitable remit, geographical boundaries or financial limits.

UK-based respondents observed that strategic decisions involve an organisation-wide effectiveness and efficiency (value-for-money) question: the choice or balance between targeting easier wins on a wider scale or investing more resources and effort on the hardest wins. Hard-win situations may involve smaller populations of animals with a higher risk of failure, but welfare is often very poor and successful interventions would make a big difference for individual animals.

Respondents did not provide specific answers to the question about decision-making criteria and process for tackling hard-win situations, although they identified four broad areas for strategic focus:

1. Decentralised decision-making on welfare interventions and a greater appetite for risk (without diluting technical capacity), acknowledging that learning occurs by giving local staff freedom to innovate and ‘space to fail’.
2. More resources invested in evidence (research and monitoring/evaluation) to gain a better understanding of the success and failure of current and proposed interventions.
3. Clearer criteria for starting equine welfare interventions in a new region, or with a new community or group of animals. These should take into account animal owners’ level of motivation and engagement from an early stage and the potential opportunities and resource availability that will enable projects to succeed.
4. A decision matrix or tool for deciding when and how to withdraw from situations where no progress is being made.

## Discussion

This international consultation on hard-wins in working equine welfare demonstrated the constraints and difficulties encountered by a large NGO aiming to improve working equine welfare in some of the world’s most marginalised communities. Results showed that the prevalence, breadth and complexity of hard-win situations were all greater than initially expected, making this an organisational priority to be addressed. Economic hard-wins occurred where animal owners or users had either an absolute lack of money to pay for equine welfare resources and services, or a real or perceived lack of economic benefit to spending money on their working animals in preference to other life priorities ([3], Fig 1). Prior to the consultation, economic factors were expected to be the most prevalent issues affecting horse, mule and donkey welfare, based on received wisdom within the working equine welfare NGO community. However, respondents also described numerous and diverse non-economic factors.

### (Recognising and addressing the complexity gap)

Despite intensive knowledge and resource inputs to its national programmes, often over many years or decades, some poor welfare situations have remained refractory to improvement. Current approaches used by working equine welfare NGOs are based on (i) strengthening equine veterinary/ healthcare services through direct provision and/or training veterinary and para-veterinary professionals and community animal health workers, and (ii) engaging with animal-owning communities to improve husbandry and work practices, using a variety of meetings, advisory services and participatory tools. Respondents described deeper, societal root causes as a common factor in refractory situations. These were thought to require a collaborative, inter-sectoral approach, rather than relatively simple, logical interventions confined to the animal welfare sector. Hard-wins in working equine welfare fit the description of complex or ‘wicked’ problems, increasingly recognised in social, economic and political contexts. They are characterised by ‘novel complexity, genuine uncertainty, conflict of values, unique circumstances, and structural instabilities’ [9–10]. Over-simplifying complex problems in international development leads to a mismatch between reality and the managerial assumptions guiding programme design [11]. Respondents to the consultation exercise recognised that assumptions about the reasoning, choices and behaviour of animal owners led to inappropriate design of equine healthcare and resource interventions. They recommended several approaches to addressing this ‘complexity gap’. Situational analysis for programming or policy decisions should include both individual and wider contextual factors and their relative contribution to welfare outcomes [3,12,13]. Situational analysis, intervention design and implementation should be undertaken by animal-owning communities and policy-makers themselves [14]. This would meet two goals: more successful equine welfare outcomes and a more appropriate allocation of responsibility between stakeholders, including owners and users, service providers, local and national governments and NGOs.

### (Lack of motivation, lack of opportunity, lack of capability)

At the end of Round 1, participants considered hard-win situations as the result of lack of motivation, lack of opportunity and/or lack of capability to improve working equine welfare. This enabled comparison with behaviour change interventions in other sectors [8] and further deliberation about how interventions could be improved. Many hard-win examples overlapped or blurred the three categories. For example, countries may have no euthanasia services because animal owners and wider society lack the motivation (for religious or cultural reasons), animal health workers lack the capability (they are not trained to carry out a procedure that society does not want) and/or the supporting organisation lacks the opportunity to introduce humane euthanasia (due to legal constraints, drug or firearm availability or staff safety).

Ellerman [9] described a move away from development assistance where helpers disseminate knowledge (capability) and incentives (motivation) to passive recipients or beneficiaries, to approaches where both the ‘thinking’ and the ‘doing’ of interventions are self-motivated by potential beneficiaries. Most participants thought that reducing direct hand-outs, while supporting owners and governments to take responsibility for working equine welfare, was critical to long-term, sustainable success. They recognised that resulting outcomes may not meet their previously preferred (‘Western’ or ‘scientific’) standards, leading to potentially difficult compromises. However, if outside experts plan and implement equine welfare interventions, they risk being poorly adapted to local conditions [9], due to unshared value assumptions, too heavily laden with the theory or ideology, equine welfare science and ethics which are not adapted to local context and they will lack unspoken ‘know-how’ about what works and what doesn’t in constrained local circumstances, and why. Failures occur because both the motivation and the knowledge are external to the ‘doers’–in this case the animal owners, other welfare actors and policy-makers. Merging ‘expert’ advice with local ‘know how’ requires extensive inter-stakeholder consultation, appropriately facilitated by a suitably experienced team who can recognise the perspectives of both parties. This role may be delivered by hiring skilled local staff who can access expert advice when needed and who can identify key community stakeholders with whom to engage. Consultation respondents said that their work was successful where lack of technical capability (knowledge) was the only barrier to welfare improvement, enabling a relatively simple training approach to solve the problem, but much less successful when lack of motivation or opportunity were also present.

### (Increasing capability—The example of migratory communities)

Participants from India, Pakistan and Nepal repeatedly acknowledged the challenge of working effectively with migratory communities or those with a high animal turnover, such as seasonal work in brick factories or at Himalayan pilgrimage sites. Wong and Regan [15] found that a continuous relationship with a health provider was important for maintaining trust, comfort and confidence in human health services for remote, rural communities. Management of chronic conditions was particularly affected by poor continuity of care; such conditions are also endemic in working equids [16–17]. Wong and Regan [15] recommended better understanding of structural barriers to healthcare continuity and ways to increase efficiency; these would also help to address hard-wins such as migratory equine-owning communities and poor retention of trained farriers. However, for people on a low income, improving the geographical availability of primary healthcare, for humans or animals, is rarely sufficient on its own to overcome other community or individual barriers. For example, the presence of a community animal health worker within or available to migratory communities is not sufficient to guarantee that animals will receive appropriate healthcare all year round; many other factors such as individual acceptability, skills, fees and competing priorities will also affect continuity of care.

### (Other strategies for improvement)

Wessells [18] emphasised the importance of the ‘First, do no harm’ principle in humanitarian aid and advocated for greater use of critical self-reflection, more specific ethical guidance, a stronger evidence base for intervention, and improved methods of preparing people coming into the situation from an outside perspective. Consultation participants identified most of these in their list of improvements to current ways of working, including the importance of interventions that do no harm to the livelihoods of resource-poor and marginalised people as well as to animal welfare. This may not sit easily with their final recommendation for a greater risk appetite in order to stimulate innovation in hard-win situations. People living in constrained situations and environments can be risk-averse in testing and adopting new agricultural or livestock-related livelihood strategies, particularly when potential risks involve productivity losses as well as gains [19]. This applies as much to owners of working horses, mules and donkeys as to other livestock.

Seeking to overcome hard-wins while emphasising long-term, sustainable equine welfare improvement involves changing the ways in which previous interventions have been implemented. In the case of working equine welfare, the breadth, scale and complexity of hard-win situations has demanded critical self-reflection and some genuine new thinking. Respondents said that inter-sectoral partnership and collaboration with human development NGOs working on livelihoods, water/sanitation and health were essential for tackling complex livelihood issues which affect people as much as their working animals. Specialist input from other sectors could also address social marginalisation, general lack of appreciation for animals in society, or gender issues relevant to working animal ownership, care and use.

A key recommendation was the need for a stronger evidence base for intervention. Schön [20] said that governments are not best placed to play the role of experimenter for the nation, identifying correct solutions and then training society in how to use them, because learning opportunities mainly occur in discovered systems at the periphery rather than official policies at the centre. The same could be said for NGOs: the role of central management is to support and observe progress and learning by (skilfully facilitated) animal owners and users at the periphery, amplify good results and use these to derive policy [9,21, 22]. Ramalingam et al. [11] emphasised the importance of real-time operational research methods in understanding complex problems and working towards improved policy and practice. A learning-by-doing approach identifies gaps between the project design or plan and its emerging outcomes, addressing these in real time as the project moves forward. This runs counter to the classic NGO plan-monitor-evaluate project cycle, or conventional research that uses pre-planned methodology sustained throughout a study. Instead, it requires the supporting organisation, whether a government department, research institute or NGO, to de-emphasise centrally-generated ‘best practice’ methods and solutions—which for many hard-wins in working equine welfare have turned out to be unsuccessful—and become adaptive, responsive ‘searchers’ rather than ‘planners’ [23]. A potential limitation of a more flexible, iterative approach is its reliance on those close to the ground having the capability to respond dynamically to changing circumstances throughout their projects. In reality, many field teams are still learning the basic concepts of equine welfare and may struggle to develop and implement even quite simple interventions. Expecting these field teams to become responsive searchers is a considerable task, particularly in new programmes or in large programmes with a relatively high staff turnover due to the high demand for scarce technical skills. Building research and critical appraisal skills in local teams is a key part of staff development but this requires—experiential learning which takes time to achieve.

### (Parallels with other sectors)

The hard-win themes identified in this consultation are not unique to working animals or the animal welfare sector. Michie et al. [8] concluded that although there are many examples of successful behaviour change interventions in (human) public health, there are countless that turned out to be ineffective. In an overview of systematic reviews of human healthcare interventions, Grimshaw et al. [24] found a very variable success/ failure rate across a wide range of contexts. Success occurred when barriers to collaboration were removed, when information transfer and learning through social influence was combined with management support, and when interventions took complexity into account, targeting multiple behaviour factors with multifaceted approaches.

Researchers working with equids and other species in high income countries also report challenges in improving welfare-related practices for both commercial and leisure animals. Improving access to welfare knowledge does not necessarily result in improved welfare management practices. Challenges may be attributed to a variety of factors. These include under-recognition by owners of welfare problems leading to delayed treatment seeking behaviour [25]; owners needing access to evidence based sources of information to facilitate decision making [26] and owners’ reluctance to change their practices despite evidence that the change would improve welfare [27].

Theories explaining behaviour change can be found in multiple scientific disciplines including psychology, sociology, anthropology and economics [28]. They include frameworks such as the theory of reasoned action, theory of planned behaviour and the trans theoretical model [29]. These models are then overlaid with additional concepts such as Cognitive Bias, which can impact the rationality of human decision making and behaviours. As noted by Weary et al. [27] when considering farm animal interventions to improve welfare, it is critical to understand the values and beliefs of stakeholders, rather than devising a purely science-based solution to a problem.

Masten [30] suggested that interventions to build resilience were more effective if focused on positive behaviours and achievement, rather than avoiding health risks and harms. The animal welfare sector is increasingly recognising the value of measuring and promoting positive welfare as well as reducing harms [31–32]. Consultation respondents from India already use participatory methods to facilitate protective processes. These include community-led tetanus vaccination campaigns, community-level equine health insurance and participatory tools emphasising the role of working equids as livelihood assets, such as ‘How to increase the value of my horse’ [14,33].

### (Suitability and limitations of the method)

The working equine welfare hard-wins consultation demonstrated some limitations described by other Delphi studies: not all participants responded to all rounds and respondents did not answer all of the five questions clearly. However, it met recommendations by Frewer et al. [34] to use an exploratory workshop to refine round one Delphi questions, use ‘cascade’ methodology (in this case cascading questions to further contacts within each country programme and requesting wider staff feedback to key respondents) and ask about a policy issue that was particularly relevant to stakeholders. Responses were enriched by the use of mixed methods (survey, semi-structured interviews, workshops) which enabled both individual reflection and group discussion inputs at different phases of the consultation.

## Conclusions, animal welfare implications and application of consultation findings

Respondents to the consultation estimated that for 40–70% of the equine populations in their programme areas (covering almost 1.5 million animals at the time of surveying), achieving sustainable welfare improvement is difficult, expensive or marginal. Participants presented a diverse range of hard-win situations, root causes and recommendations based on long-term, extensive experience in the field. All respondents agreed that if equine welfare improvements are to span generations of animals, programmes need to embed good welfare capacity, motivation and practical application in all relevant actors and cannot rely on quick-wins alone. Examples of quick wins include disaster relief, creating infrastructure such as shade shelters and water troughs, euthanasia compensation schemes, vaccination and deworming programmes and distribution of free grooming kits.

In the three years since this consultation was carried out, several organisational initiatives have addressed the issues it identified. These are intended to facilitate national programmes to investigate the root causes of problems in detail and engage local stakeholders in developing interventions that are appropriate to their infrastructure, constraints and cultural norms.

An Innovation Fund enables Brooke staff, partner organisations and third parties to address local equine welfare issues through small-scale trials of peer-reviewed ideas. This encourages flexible programme design and aims to remove the fear of failure that previously hindered new initiatives. Innovators complete a simple post hoc reflection to inform a decision on whether to scale up the innovation, and enable internal and external sharing of both positive and negative lessons learned. Initiatives funded include trial collaboration between Brooke and an international development NGO to incorporate equine welfare topics into existing livestock owner health and welfare capacity building activities.

Additional research capacity supports robust, context-specific, applied research generated at local level. Success has already been seen in the form of completed research projects investigating animal owners’ and service providers’ motivations and constraints.

A new Monitoring, Evaluation, Accountability and Learning framework aims to ensure a collaborative approach to generating evidence within a project cycle, between the organisation and its beneficiaries. This encourages local teams to recognise when to adapt programme activities according to interim findings, rather than waiting for an evaluation at the end of the project cycle. The framework includes testing indicators linked to the Department for International Development’s Sustainable Livelihoods Framework to enable both economic and wider livelihoods-related changes associated with an intervention to be measured and monitored.

There is increased emphasis on phasing out direct veterinary service provision in order to reduce a handout culture and avoid crowding out local service providers.

A cost-benefit analysis framework has been developed to address the trade-off between tackling relatively easy, large scale and less acute welfare problems and smaller-scale, more acute, trickier problems with a higher risk of failure. This informs decisions on programme planning in existing national programmes, including development of consistent exit criteria, and assessment of potential new countries of operation.

Ultimately, the organisational changes made in response to the hard-wins consultation have a single common aim: to optimise sustainable welfare improvements for working equids within the resources available.

## Supporting information

S1 Table Hard wins Round 1 questionnaire. The questions posed to participants in round 1.
(DOCX)

S1 File Hard wins Round 1 response Country A. (DOCX)

S2 File Hard wins Round 1 response Country B. (DOCX)

S3 File Hard wins Round 1 response Country C. (DOCX)

S4 File Hard wins Round 1 response Country D. (DOCX)

S5 File Hard wins Round 1 response Country E. (DOCX)

S6 File Hard wins Round 2 conversation summary. (DOCX)

S7 File Hard wins Round 2 Appendix 1 summary examples. (DOCX)

S8 File Hard wins Round 2 Appendix 2 summary opportunities. (DOCX)
