# Supplementary material for: Improving working equine welfare in ‘hard-win’ situations, where gains are difficult, expensive or marginal
Source: PLoS One. 2018 Feb 6;13(2):e0191950. doi: 10.1371/journal.pone.0191950 (PMC5800664; doi:10.1371/journal.pone.0191950)
Supplement: S1 Table — The questions posed to participants in round 1. (DOCX) [file pone.0191950.s001.docx]

Hard Wins Round 1 Questionnaire

| Question no. | Purpose | Question(s) |
| --- | --- | --- |
|  |  |  |
| 1 | Presence and nature of problem, root cause(s) | Have you identified any 'No-win situations' in your work? If so what are they, and what is the underlying root cause? |
|  |  |  |
| 2 | Impact | How big an impact have these 'No-win situations' had on the programme's work? (considering the number of equids affected, the magnitude of suffering and programmatic efficiency/ effectiveness) |
|  |  |  |
| 3 | Nature of current approach(es), effectiveness | What did the Brooke do to address these situations and what were the results (good and bad)? |
|  |  |  |
| 4 | Alternative approaches, with reasons | What other ideas and suggestions do you have to deal with these 'No-win situations', and how will these approaches be more effective? |
|  |  |  |
| 5 | Improving strategic decision-making | How can the Brooke make more consistent decisions about how programmes tackle these 'No-win situations'? |
|  |  | ~ Are there certain decision-making criteria that should always be considered? |
|  |  | ~ Could a decision-making process be mapped out based on such criteria?If so, how does this account for very different situations? |
|  |  | ~ When should these decisions about No-win situations be taken: when scoping new countries, when scoping new areas within a country, once working in an area, as part of a strategic review….? |
|  |  |  |
