# Supplementary material for: Improving working equine welfare in ‘hard-win’ situations, where gains are difficult, expensive or marginal
Source: PLoS One. 2018 Feb 6;13(2):e0191950. doi: 10.1371/journal.pone.0191950 (PMC5800664; doi:10.1371/journal.pone.0191950)
Supplement: S1 File — (DOCX) [file pone.0191950.s002.docx]

- Have you identified ‘No Win Situations’ in your work, and what do you think the root cause of these are?
  - - Inadequate consideration of equine welfare in the development policies, strategies and plans of the government. This is because of the focus of government development policies and strategies only on major areas that are supposed to have direct effect on poverty and social changes (development issues); and equine welfare per se is not an issue for the government .Equids are not yet considered as significant contributors to development.
    - Lack of adequate action from urban equine owners towards improving the welfare of their animals. This is because of poverty and the low income of the owners that are solely dependent on their equid. The priority is to get as much immediate income as possible with out adequate consideration for the welfare of the animals. For example, the existence of only one major market day in a week in each region makes equine owners to use their equine exploitatively by overloading, overworking (working the whole day), and inadequate care. Another example, owners could not influence and afford to access improved cart design and farriery services (non-injuries and equine friendly); as they could not afford the cost associated with the improved services.
    - Lack of resources (water, feed, shelter, friendly husbandry materials) by owners. This is mainly because of low income of the owners and unavailability of some resources (e.g. water in key towns) within the reach of the owners.
- How big an impact have these ‘No Win Situations had on the programme’s overall impact?  (considering both the number of equids affected, the magnitude of suffering and programmatic effectiveness/efficiency).
  - - The impacts of these situations may not be so significant; but would be big challenges for the Brooke programme and need huge efforts. They may also challenge the sustainability of the programme. Together with the Brooke interventions, other ongoing development works by other actors will also help address some of; e.g. infrastructure development (water supply structures, roads, etc.).
- What has The Brooke done so far to address these situations, and what have been the results: good and bad?
  - - The Brooke has been involved in a lot of awareness raising/attitude changing, government influencing and capacity development interventions to address the above situations. The Brooke has also supported communities to get better access to basic needs of the equid (e.g. water supply and feed supply). Encouraging results have been achieved, but bringing ultimate change needs more time and more efforts.
- What ideas, suggestions do you have to deal with these No Win Situations and why do you think these suggested approaches will be effective? (This could be as radical as not doing anything.)
  - - The Brooke should strengthen and continue its engagement on awareness raising, attitudinal change and government policy and practice changes.
    - The Brooke should strengthen the intervention to link equine welfare with livelihoods as a way of showing and convincing the government how improving equid welfare can contribute to development.
    - The Brooke should invest on the improvement of key equid needs (e.g. water, shelter), though this requires a huge financial investment and technical (engineering) work.
- What does the Brooke need to do so it can tackle these ‘No Win Situations Better’? e.g.
  - Be more innovative?  (with examples of innovative ideas?)–Work with existing government system and grassroots structures in the country
  - Be more flexible in approaches to communities – not a one size fits all approach?– Interventions to address key gaps in equid welfare (e.g. Capacity building on water and feed supply in the country).
  - Work more closely with different actors (advocacy, community influencers) – or build better relationships, understanding with communities? - work very closely with local government authorities and government service providers, influencing work at central government level.
  - Work with other organisations to tackle wider issues that impact on welfare (e.g. domestic violence) and if so how does the Brooke make sure it stays focused on its mission with such an expansive approach? - Mobilization and collaboration with equine focused Non-government organizations within the framework of the defined strategic objectives and approaches of the Brooke.
  - Should we be tackling these no win situations at all – should we just focus on the areas we can make a difference

We should be tackling the situations if we are to achieve a sustainable change on equine welfare. As life is full of challenges and opportunities challenges should not make us stop our efforts and we should look forward through all possibilities.
