# Supplementary material for: Improving working equine welfare in ‘hard-win’ situations, where gains are difficult, expensive or marginal
Source: PLoS One. 2018 Feb 6;13(2):e0191950. doi: 10.1371/journal.pone.0191950 (PMC5800664; doi:10.1371/journal.pone.0191950)
Supplement: S2 File — (DOCX) [file pone.0191950.s003.docx]

**Have you identified ‘No Win Situations’ in your work, and what do you think the root cause of these are?**

Region 1 project:

*-The administering of vitamins to ‘help’ the equine is an almost cultural practice, they have been administered to cattle, pigs and even fowl for a long time. The inclusion of horses within this ‘care’ is a progress from the point of view of recognizing the equine. It is something that they like and will continue to do, although we won´t provide/facilitate them.*

*-The control of tick infestation which is so difficult for equine owners due to the environment, especially in key warm/wet months when the infestations are tremendous.*

Region 2 project:

*-The precarious economy of many owners and the inequality of belongings/property (specially limited access to land to cultivate)into the communities means that they overwork and overload their equines for example: a producer of a certain crop contracts three people (owners) with his equine to take the harvest 2 kilometers distance and he pays them $x per trip, the owner has to work faster with his equine and whilst more trips a day mean more gain, what he earns only covers the subsistence expenses of his large family.*

*-A hand out culture has been formed in the communities by two situations that happened in the past, first the acute natural disaster of xxxx and finally the political unrest in xxxx. After these two situations many international organizations came to bring help to the affected communities without educating them. The problem is that these days any organization that brings them training and advice is not well received because it is always expected that they will give away things. Also the Government of this country supplies help in products and money to the families of low income in the communities instead of training them to create more work for subsistence.*

- **How big an impact have these ‘No Win Situations had on a programme’s overall impact?  (considering both the number of equids affected, the magnitude of suffering and programmatic effectiveness/efficiency)**

Region 1 project:

*-With respect to the vitamins it does not have an impact. Possibly to deny the service could have a negative impact.*

*-We as an organization can only teach equine owners how to prevent and control tick´s infestation but not how to eliminate the problem.*

Region 2 project:

*-The low participation of the owners at the training activities affects the welfare of the equines because they do not learn care practices for the equines.*

*-some owners that participate have not put into practice what they have learned and are not interested in carrying them out and their equines continue to suffer, they justify themselves by saying that they don’t have time or resources etc.*

*-The owners don’t want to pay for the services that we bring to their equines, (preventative treatments, nutritional supplements, hoof care) and for this reason in many communities the local community animal health care agents and the service providers do very little work with the equines.*

- **What have you done so far to address these situations, and what have been the results: good and bad?**

Region 1 project:

*-It has been explained that there is necessary to provide a balanced diet that contains enough calories on daily basis, however, they continue demanding the vitamins.*

*-A sustainable method was investigated for the control of the ticks (salt water baths). We trained equine owners on the subject and some of them have adopted this practice.*

Region 2 project:

*-Teach the participating owners care practices for the equines that do not depend upon service providers of services within the community and that they are the ones who resolve their problems.*

*-Teach the whole family care practices, the kids and the wives are now responsible for cleaning the living area of the equine.*

*-Visits to the homes have been carried out to train the owners and their families, also, we have done some visits to the working areas*

- **What ideas, suggestions do they have to deal with these No Situations and why do they think suggested approaches will be effective? (This could be as radical as not doing anything.)**

Region 1 project:

*-They don’t want to stop providing vitamins; they have the perception that it is good for their equines*

*-The idea that they have is that they should fumigate or treat the problem only when they see the kind of ticks that correspond to a massive infestation, they think that until that point they don’t have much of a problem*

-They usually are requesting us materials or medicines for free because they say that they don´t have enough resources to buy them and follow-up our recommendations.

-They usually complain because we don´t give free medicines and materials as other organisations do.

- **What does the Brooke need to do so it can tackle these ‘No Win Situations Better’? e.g.**
  - Be more innovative?  (with examples of innovative ideas?)
  - Be more flexible in approaches when working with communities – build better relationships, understanding with communities and really tailor work to what suits each community?
  - Work more closely with different actors (advocacy, community influencers)? How will this help these ‘no win situations’?
  - Work with other organisations to tackle wider issues that impact on welfare (e.g. domestic violence) and if so how does the Brooke make sure it stays focused on its mission with such an expansive approach?
  - Should we be tackling these no win situations at all – should we just focus on the areas where we can make a difference?

Region 1 project:

*-Train in nutrition but including practical exercises in each community measure results that are palpable such as: choose 5 equines from a community and provided them with a cheap energy supplement like corn mixed with water and salt, during a month and measure the progress of these equines against another group of ‘vitamined’ equines. Then make a comparison which could include variable of weight (only as an example; but the methodology would be indicated, the people should react to the results.*

*-I wouldn’t say be more flexible, I would say be more ‘horizontal’; work on what is possible but that it be things that the people from the community would be interested in; not impose criteria without listening to what they say and according to this plan and design the work.*

*-With respect to the ticks we should continue to look for alternatives.*

Region 2 project:

*-Create alternatives that are easy to replicate and that are sustainable for the owners, e.g. Making of hoof picks with local materials.*

*-Work in the communities based on the needs and be flexible in the working methods with the communities – establish better relationships with the communities with the understanding that trust and relationship is key for success*. *This is the most appropriate because the work with the communities is not a cooking recipe, you need to implement different methods and these vary from one community to the other. What assures the success of the activities is to carry out horizontal programs, this means accommodating the needs of the community to the goals of the local organization so that the community sees that we are working for the good of them and their equines and not accommodating the goals of the local organisation.*

*-We should focus on areas which we can make a difference for the welfare of working equines.*

*-In this country, to work with other communities is not easy because they have their own objectives and usually are not opened to accept new ideas.*

*-The government institutions have a lot of limitations in terms of resources to work at the field, due to this, when you try to engage with them, they usually want you to provide resources so they are able to do their activities.*

***-Dependency and poverty*** *are the main issues in the communities. Income inequality is very big in this country, even into the communities. The government doesn´t have the capacity to address these issues due to corruption, political influences, etc. so, for the local organization it would be very difficult to address these issues because they are structural problems.*

*In summary, there are four “No wins situations”:*

1. *Stop vitamins provision in region 1*
2. *Solve tick´s infestation in region 1*
3. *Improve economy in rural communities in region 2*
4. *Change the culture of dependency and gratuity in general*
5. *Work in coordination with other organizations in general*
6. *Lack of efficiency and transparency of government institutions in general*
