# Supplementary material for: Improving working equine welfare in ‘hard-win’ situations, where gains are difficult, expensive or marginal
Source: PLoS One. 2018 Feb 6;13(2):e0191950. doi: 10.1371/journal.pone.0191950 (PMC5800664; doi:10.1371/journal.pone.0191950)
Supplement: S3 File — (DOCX) [file pone.0191950.s004.docx]

1) Have you identified any ‘No Win Situations’ in your work? If so what are they and what is the underlying root cause?

a) In this country: As The Brooke is not giving anything for free (materials, medicine, vaccinations etc) there is almost no interest from the owners’/users’ sides to join the educational trainings/meetings. Cause: population’s perceptions are ‘spoiled’ by last national and international policies of free assistance (i.e. if you want us to improve/change this and that, give us the following things… because we think/feel/know/have been told… that these are the basics in order to achieve the changes you want)

b) If the price for a new animal is within the range of ‘economically affordable’ (i.e. relatively low) for owners, summing up all spending for improvements (e.g. medicines, environment, more time for rest which means less immediate income etc) must not surmount the cost for a new equine.

2) How big an impact have these ‘Now Win Situations’ had on the programme’s work? (considering both the number of equids affected, the magnitude of suffering and programmatic effectiveness/efficiency)

Impact so far: influencing owners/users and thus a positive welfare effect on equines takes longer than expected. Longer term effect not yet known as programme young, lack of back up experience

3) What has The Brooke done to address these situations and what were the results?

1. change of strategy in communicating with communities/owners, results seem to improve quantitatively
2. not addressed yet

4) What other ideas and suggestions do you have to deal with these ‘No Win Situations and how will these approaches be more effective?

In a few months from now, with more data, strategy will be reviewed

5.       How can The Brooke make more consistent decisions about how programmes tackle these ‘No Win Situations’?

Are there certain decision making criteria that should always be considered? Each programme could explicitly name its country specific ‘no wins’ in new or annual planning in order to be aware of such criteria/situations.

Could a decision making process be mapped out based on such common criteria? If so how does this account for very different situations? The only recommendation I see for a ‘mapping’ is: watch out for the specific ‘no wins’ in countries/programmes in order to consider them early enough in your planning (contingency of tackling)

When should these decisions about No Win Situations be taken: when scoping new countries, when scoping new areas within a country, once working in an area, as part of strategic review? In all of those i.e. whenever plans or decisions are due.
