# Supplementary material for: Improving working equine welfare in ‘hard-win’ situations, where gains are difficult, expensive or marginal
Source: PLoS One. 2018 Feb 6;13(2):e0191950. doi: 10.1371/journal.pone.0191950 (PMC5800664; doi:10.1371/journal.pone.0191950)
Supplement: S4 File — (DOCX) [file pone.0191950.s005.docx]

This is an interesting and important conversation topic. I intend to pen down my thoughts as they come to me and leave it for you to look for *ratio decidendi.*


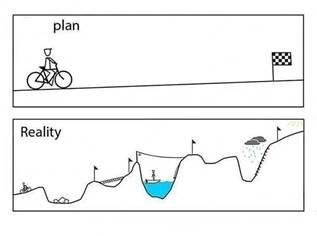


There are two kind of no win situations that we usually encounter in context to Brooke work, one existing naturally in reality before Brooke interventions and other is Brooke perception of what should have been…… in latter case, it is our limitations in capturing change via application of available measurement tools, current tools are designed to measure animal based indicator for ring fenced population of animals, in our case they are applied to fluid and varied situations, where animals are being changed/exchanged along with owners/users coupled with the phenomenon of fragmenting communities. Measurement tools at best show us long term trends, but are not capable of capturing short term or intermediate results. Change is taking place all the time but we cannot capture it through our methods. There is need for a more flexible approach that opens up new venues of capturing the impact of interventions.

The usual approach adopted by the Brooke is to identify and endeavor to remove the cause of the problem. Sometimes it is not possible because the cause cannot be found; because there are too many causes; or because the cause is human nature and cannot be removed. In such cases we are usually paralyzed, and the problem cannot be solved by more analysis. There is a need for an approach/design for a way forward that takes into account these in build limitations – leaving the cause in place. It is like designing a Fuzzy logic camera which takes into account the shake of the hand yet produces perfect picture.

Another important aspect is lack of clarity about the concept of welfare vis-à-vis husbandry efforts at hand. How can limited husbandry efforts i.e. farriery, harness, water and relieving pain can improve welfare i.e. psychological health of animal when many other risks are still not known despite aggressive community work, under such circumstances how can one plan a specific intervention on a moving animal population, Brooke could land up doing more harm than benefit the animal in absence of crucial information.

Human behavior greatly impacts the equines welfare. The myths and practices learnt over a period of time cannot be changed in a short span. This calls for patience and determination. Deliverables are not always achieved, and incremental changes are missed by the radar.

Institutional take on equine is marginal or nonexistent, this further aggravates the situation. The Brooke can better tackle “No Win Situations” by evolving pragmatic and flexible program policies sensitive and more responsive to issues of each area rather than developing a blanket cover for all issues. Effectiveness framework guidelines in its true spirit demands taking on board the different stakeholders and leverage the program through different program components for working equine welfare.

Above requires involvement of relevant stakeholders at appropriate stage of the program/project to ascertain more about the risks to animals to improve the context of Brooke husbandry advices to owner/users.
