# Supplementary material for: Improving working equine welfare in ‘hard-win’ situations, where gains are difficult, expensive or marginal
Source: PLoS One. 2018 Feb 6;13(2):e0191950. doi: 10.1371/journal.pone.0191950 (PMC5800664; doi:10.1371/journal.pone.0191950)
Supplement: S5 File — (DOCX) [file pone.0191950.s006.docx]

Have you identified ‘No Win Situations’ in your work, and what do you think the root causes of these are?

Programme in this country has not been going for long enough to have real experience yet of a no-win situation

- No economic gain:

If users, not owners, do they care if animal dies? They can get another.

Discussed drivers in one region – although the evaluation data is limited, there does appear to have been **some** gain. It certainly seems to have taken a longer time to get lower level of improvement than, for example in rural areas. So not a “no-win”.

- Cultural constraints:

There could be a no-win situation in rural areas if we come up against a very resistant traditional leadership. Would be very difficult to break down. But if leadership pro Animal Welfare – good ally. In an urban environment, less traditional hierarchical structures. But possibly more difficult to get a social “norm” pressure to treat animals well.

- Logistical constraints:

If seasonal workers and very mobile, can we reach them? (In one region the seasonal workers’ horses are actually in better condition. It will be interesting to see in another region with the transhumance each year.)

- Communication constraints:

Do we bring about some no-win situations by not being clear enough what we do? Elitist, conceptual approach? Have we made what we do practical enough, clear enough, justified enough? Do we give people a win-win “offer”? (example of group was using a better halter – have we made the “why” clear enough?)

Do we have a strong enough overall “why improve Animal Welfare” message?

“xxxx” in local language is possibly too close to “wellbeing” in English – with a suggestion of comfort. Difficulty in finding a word to translate the concept of welfare which is beyond protection from cruelty and captures sense of basic welfare.

- “Mission” constraints

If we work with non-animal welfare organisation, they may not have required level of interest. If we can stimulate the interest can be leverage to get more allies. If we can’t, could become a no win situation. If communities are interested, partner organisation could become barrier rather than facilitator.

How big an impact have these ‘No Win Situations had on a programme’s overall impact? (considering both the number of equids affected, the magnitude of suffering and programmatic effectiveness/efficiency)

Yes would be important if we find working with drivers is a no win.

What has The Brooke done so far to address these situations, and what have been the results: good and bad?

What ideas, suggestions do you have to deal with these No Situations and why do they think suggested approaches will be effective? (This could be as radical as not doing anything.)

Following are our ideas on how to address, rather than experience of what we have done:

- Economic argument is key as we target “working” animals
- However whilst economic argument is the single most important argument, it is not the only one
- Also, within the economic argument there could be more angles to develop – e.g. “time preference” concept used in health economics – how to elicit choices and perceptions in relation to smaller investment time/money now vs. higher time/money costs later
- Social norms and peer pressure can play a role – linking our advocacy at policy level with communications for influencing at local level with community engagement strategies for raising awareness, changing behaviours etc.
- Also the type of relationship that exists between man and horse. The one region evaluation talks about the type/quality of the relationship playing a role; can we improve the user sense of mutual relationship and dependence with his horse
- Type of relationship and mutual responsibility of owner / user – can we work with both together and separately to develop responsibilities / dependencies between horse / owner / user?
- Can we use examples where people are totally dependent on animals – e.g. donkey ambulances – outside their normal day to day lived experience?
- Also self-perception / dignity – many poorer families will take pride in presentation of their children; maybe they have the same pride with their animals, especially if not a financial cost
- How to get routines established as non-special, daily part of life – e.g. washing a donkey not something that amazes
- Identify and support “champions of Animal Welfare” / “change agents”, work out how we can best use them as part of our community engagement strategy
- Understand barriers in local context – customary beliefs / traditional practices that will constrain us – and assess who / how we can overcome them or if will be no win situation. Are there parallels in other sectors? Gender for example – traditional practices in relation to girls’ schooling – changed by working on policy and community awareness
- Do we offer enough - other than advice and training and awareness-raising; make sure there are “wins” which secure interest in and commitment to Animal Welfare. Combining tangible benefits to equines with tangible benefits to humans through Income Generation Advice for example.

How can the Brooke make more consistent decisions about how to tackle these No Win Situations in Programmes?

Using Monitoring and Evaluation systems / acceptable level of change – can make decision that not making any / enough progress given time and cost. BUT need to be realistic about time. And need to be realistic that quick win may be a quick lose too – i.e. results look great but go back in a year or two and little left.

Ensure we learn proactively and share learning.

Ensure we allow and learn through failure and allow innovation which may be higher risk to tackle serious welfare issues in what seems a no win situation.

Once we know what really are dead-end definite no-win situations, let’s be sure we all avoid them.
