# Supplementary material for: Improving working equine welfare in ‘hard-win’ situations, where gains are difficult, expensive or marginal
Source: PLoS One. 2018 Feb 6;13(2):e0191950. doi: 10.1371/journal.pone.0191950 (PMC5800664; doi:10.1371/journal.pone.0191950)
Supplement: S6 File — (DOCX) [file pone.0191950.s007.docx]

**Strategic Conversation Summary: No Win Situations.**

*Participants: Senior representatives from seven Brooke Offices*

**What are no win situations**? No win Situations were initially described as ‘*Situations where Brooke cannot improve welfare because this brings no socio-economic benefit for owners or users*.’ Everyone consulted felt this should be expanded firstly because Brooke could always do something to improve welfare. Secondly there are many other examples and explanations for Brooke’s work ‘not winning’. So although a lack/perceived lack of socioeconomic benefit is a barrier other barriers, such as a lack of resources/opportunity, are equally important. Therefore the real challenge is the hard win situations; where welfare improvement is difficult, expensive and marginal.

**Why do hard win situations exist**? *(Please refer to annex 1 for full details)*

A number of underlying factors were identified that made Brooke’s work less effective at improving welfare. This includes working:

- With owners/users that see no economic or social benefit from caring for the equine (misuse, abandonment)
- With actors with no empathy for equids or animals
- With communities with deep seated social issues; e.g. drug/alcohol/solvent, or working illegally; who are not interested or are afraid to engage in projects
- In areas with a high animal turnover and with migratory communities (high turnover of people)
- With owners with so few resources they are unable to care for their equid or cannot appreciate the longer term benefits of caring for their animal better. (A low time preference)
- With private service providers or government vets with no financial incentive to treat equids correctly
- With people who will not cooperate even though benefits are mutual, especially peri-urban/urban communities
- In unsafe areas where interventions cannot be delivered and teams cannot be supported

In addition to these external factors there were many insights about how Brooke’s own ways of working actually created or exacerbated hard win situations. These are particularly interesting because if addressed Brooke may have an opportunity to tackle hard win situations more effectively in the future.

**What Brooke ways of working could create or potentiate hard win situations?**

1. Brooke programmes struggle to design interventions that are most likely to improve key welfare issues and their underlying causes. Therefore welfare improvement is limited and hard win situations are created. A number of reasons were suggested for this which, if addressed, could improve programme effectiveness:
   1. Improving equine welfare is inherently difficult in countries where welfare is unheard of and people/animals live in harsh environments with few resources. Teams have a daunting task and work in isolation as animal welfare is rarely a priority for local people, governments or organisations.
   2. Brooke teams struggle to identify and tackle the root causes of welfare problems. Many interventions improve welfare by changing key actor behaviours and practices. Therefore teams must understand what drives or prevents certain behaviours and interventions must address these barriers. This is not easy. Teams have to understand welfare, veterinary and human behaviour change concepts and theses skills are not readily available in-country. Instead a small UK team trains programme staff in complex scientific subjects through a few short visits and limited distance support. Yet programmes are still expected to design/deliver complex interventions and the quality can be compromised.
   3. UK and programme teams have mixed understanding about what the Brooke is trying to achieve. Should teams alleviate immediate suffering, e.g. free treatment or providing resources? Or should teams focus on changing behaviour so key actors improve equine welfare themselves? Currently programmes grapple with both but as short term alleviation often undermines more sustainable interventions this can make activities ineffective. e.g. free treatment undermines the development of local service providers. A second conflict is whether programmes should work through local actors when the Brooke could have a much greater impact working directly? Is the Brooke prepared to accept the slower or reduced welfare improvement when working through local actors? The current strategy does not clarify Brooke’s position in these critical areas creating confusion and occasionally conflict in teams.
   4. Brooke reporting currently focuses on coverage, uptake and activities completed rather than the changes made to welfare and target actors. Therefore there is no recognition or reward for programmes to make their interventions more effective. Furthermore programmes may not even recognise when they are not being effective as coverage/uptake does not guarantee welfare improvement. If Brooke wants to be more effective perhaps different questions should be asked of programmes.
2. Limited flexibility innovation at field level. Tackling hard wins requires innovation and flexibility. Field teams need the confidence, flexibility and support to try new things and respond quickly to changing environments. However innovation comes at the risk of failure. If Brooke wants to culture innovation it must embrace the increased risks associated with it.
3. Inherent challenges of Brooke’s mission. Actors are more likely to treat equids well if they understand and value animal welfare principles. However it is very difficult to teach welfare using only one species, as rationally it applies to all. Would Brooke be more effective if welfare skills were developed for other animals alongside equids? Additionally most service providers cannot earn enough from equine treatments and rely on livestock revenue streams. Could Brooke create more sustainable service providers by developing their skills to treat livestock, alongside equids, to diversify their income and create sustainable businesses?

**Should Brooke tackle or avoid no/hard win situations?**

Everyone felt hard wins should and could be tackled. Quick wins alone will not generate welfare improvements that span generations and the quality of welfare improvement was a priority. Tackling hard wins may also trigger teams to review their approaches catalysing improvement and innovation. However many accepted that some hard wins maybe too expensive, time consuming or risky to tackle. Brooke must recognise these situations early and make timely decisions; to either tackle differently or withdraw; so resources can be used more effectively. This caused some conflict as people were uncomfortable walking away from these scenarios as they often involve the neediest equids. Instead some felt that Brooke should provide palliative care alleviating suffering; accepting this could not be sustained. Others felt this may make these challenging situations harder by engraining negative behaviours. All parties wanted the next strategy to provide guidance on this. Everyone agreed that decisions should be transparent and evidence based. Everyone also stressed the importance of honest communication, internally and externally, sharing the real reasons and evidence behind decisions.

**Opportunities to tackle hard win situations more effectively in the future:** *(please refer to annex 2 for more details)*

1. Brooke needs to clarify organisational priorities so teams (UK and Programme) have greater direction when making decisions about hard win situations. Areas requiring particular clarity include:
   1. Should immediate suffering always be alleviated or is Brooke prepared to accept suffering in the short term for greater benefits in the future? If so to what degree?
   2. Is Brooke prepared to sacrifice the quality of interventions in order for them to be sustained? If so, to what extent?
   3. Should Brooke prioritise equids in greatest need, whose welfare is very difficult to improve? Or equids in better condition, whose welfare Brooke can improve more easily? What is more important improving very poor welfare marginally, or improving poor welfare dramatically? Should Brooke try to avoid hard win situations or target equids in the greatest need?
2. Brooke must clarify decision-making responsibility in hard win situations; ensuring timely decisions are based on evidence and communicated transparently. Brooke should be confident sharing information about challenges externally as this may enhance Brooke’s credibility with supporters.
3. Brooke should encourage programmes to focus on the quality of welfare improvement rather than coverage/uptake and completing activities. Focusing reporting and discussions on the changes being made to animals/actors is critical. However this will only be meaningful if it forms a basis for decision making. e.g. could a major criterion for internal expansion be a programme’s ability to improve welfare effectively?
4. Recognising that Brooke’s mission is inherently difficult could Brooke make more use of:
   1. Advocacy: to create an enabling environment for welfare change. In some hard win situations perhaps advocacy is the priority/only option instead of field work.
   2. Partner organisations: Could Brooke develop more effective partnerships with other organisations to tackle wider social issues that are beyond Brooke capabilities? A real challenge is finding interested partners who can/will conform with the current partnership and reporting requirements. Brooke may have to discuss its mission more broadly to attract partners; e.g. in the terms of livelihood generation, healthcare development, empowering women, and accept welfare may be an additional benefit for partners, not the focus. Alternatively Brooke maybe more effective working with partners with an interest in welfare; accepting that wider social issues are beyond the organisation’s capability. Finally Brooke should review whether it is effective to cascade very technical interventions such as service provider training or direct treatment to partner organisations when Brooke struggles to develop skills within its own teams. Perhaps Brooke would be more effective delivering these technical interventions directly?
   3. More varied interventions and activities: Much of Brooke’s work relies on capacity building external actors. This assumes people behave as they do because they know no better. In reality there any many other barriers to good behaviours; especially a lack of motivation and a lack of resources /opportunity. Could Brooke do more to address motivation and create opportunities/resources in countries? Such as microfinance schemes for owners, business start-ups for private service providers, improving drug/equipment and local resource availability e.g. water, shelter? (rather than relying so heavily on actor capacity building)
5. Could Brooke better support and develop programmes’ capabilities to design and deliver effective interventions:
   1. Develop technical skills through the secondment of head office staff; particularly in new programmes. Plan start-up strategies for new programmes that limit technical activities until capacity is developed. Increase the use of technology to deliver distance training (this will require better internet connections in country, and better commitment from in-country staff.)
   2. Develop stronger strategic planning and programme management skills in programmes so they can develop a rational theory of change and plan interventions strategically and logically.
6. Would Brooke consider developing wider animal welfare and/or veterinary skills in actors if this would lead to more sustained and engrained behaviour change? OR would this lead to mission creep and stretch programme skills too far?
7. Could Brooke’s programme model look significantly different? With the advent of mobile healthcare (healthcare through phones/handheld devices) could Brooke actually create infrastructure to technically backstop service providers in the field or link services to owners etc? Does Brooke need to exit an entire country or could it achieve countrywide coverage by creating equine service providers at field level (using external actors) then continuing distance support from a national hub? (This could be a self-sustaining fee-for-service business)
8. Could Brooke foster more innovation within the organisation, particularly in-country. Is Brooke prepared to take the increased risk associated with trying new things? Could decentralised decision-making support innovation or will it dilute the technical capacity needed for effective intervention design?
