# Supplementary material for: Improving working equine welfare in ‘hard-win’ situations, where gains are difficult, expensive or marginal
Source: PLoS One. 2018 Feb 6;13(2):e0191950. doi: 10.1371/journal.pone.0191950 (PMC5800664; doi:10.1371/journal.pone.0191950)
Supplement: S7 File — (DOCX) [file pone.0191950.s008.docx]

| **No/Hard Win Situation**  *Situations where Brooke has struggled to improve welfare* | **Potential root causes/or reasons why Brooke struggles to make an impact** | **Suggested alternatives/Additional Info**  *(Please note this is not exhaustive)* |
| --- | --- | --- |
| **‘Situations where owners either cannot or are not motivated to make changes to improve equine welfare’** | | |
| Brooke has struggled to improve the way people treat equids they rent but do not own. Examples include:   - People renting animals in Brick kilns India/Nepal - Tour guides renting equids to carry tourists up pilgrim sites (India). - People renting equids in Ethiopia and Kenya | - There is no economic benefit for users to improve everyday welfare as do not benefit from longer term health improvements. Their priority is to earn as much as possible that day, tomorrow they may have a different equid. - This is underpinned by a lack of empathy, and appreciation for animals in society. | - Analyse the situation carefully to identify the root cause(s). Don’t make assumptions about what drives behaviour, then target activities to address the root cause. - Look for alternative actors who could influence the behaviour of these users   - E.g Work with actual owners who let out the equids. The owners will benefit if their horses are treated better by those renting them. The people letting out the equids may be able to influence the behaviour of the users renting them by only renting to those who look after the well. - Accept that users will never look after equids well and offer alternatives to equids. (bike/car/bus). This may result in equids being abandoned so interventions should be prepared. - Policing/restrictions/fines for abuse - Consider whether it is possible to work with owners with no interest or motivation. Recognise and accept when interventions are not being effective and consider exit if all other options have been exhausted. - Target younger generations and build welfare, empathy and raise wider awareness about equid welfare. Can welfare really be taught when only discussing one animal? |
| Occasions when owners physically are not able to improve their equid’s welfare because of external barriers. e.g.   - Brick kilns – working conditions are often dictated by the brick kiln owner. High brick quotas encourage owners to overload, water points not available to offer water etc. - Lack of resources (money, food, water) Resources so limited that there is no option but to prioritise human need over equine. - Lack of healthcare infrastructure   It is impossible for equine owners to seek treatment for diseased/injured equids. | - Owners are often the key actors in programmes however they are not always able to make the changes needed to improve welfare. | - If owners are not in a position to improve welfare are there other actors who can create a more enabling environment e.g. Involve higher powered actors (brick kilns owners, regional national government, other NGOs) - Can Brooke help remove infrastructure and resource barriers by improving access to important resources? e.g. water points in Ethiopia, water points in brick kilns, drug and equipment availability (drug revolving funds) - Can Brooke create healthcare providers where they don’t exist?   - Are equine only service providers sustainable in a privatised system? Can they earn enough money from treating equids alone? - If not, they are unlikely to sustained. Could Brooke be setting up community based healthcare workers who could treat other livestock species as well as equids, to address this issue?   - Is Brooke prepared to take the risk of setting up alternative healthcare systems? Who will train and regulate service providers after Brooke exits?   - Is it legal to informally train service providers? |
| Working in communities with deep seated social issues such as drug abuse, alcohol abuse, solvent abuse, or using equids to work illegally.   - Unable to do activities due to interruptions, poor attendance. People unwilling to meet if working illegally. | - Brooke does not have the expertise to operate in these difficult situations, so it is very difficult to deliver interventions | - Could Brooke with partner organisations with more experience address the social issue in hand? Or does this risk programmes getting very distracted from the welfare mission. - Should Brooke be working in these areas at all? If not, could they be identified earlier so programmes can exit or not enter. |
| Owners see no value in caring for the equine as when it is sick/dies. The cost of replacing it is cheaper than cost of caring for the sick or injured animal.   - In some countries owners may be wealthy enough to replace equids easily - Brick kilns in south Asia   Equids bought very cheaply because they are sick/diseased and worked until they are can do no more | - No economic motivation to improve welfare - Underpinned by a lack of empathy, and appreciation for animals (and in particular equine animals) in society. | - Can Brooke build wider empathy for equid welfare? Is it possible to build/train/develop welfare when only discussing one animal? Welfare as a concept applies to all animals. Should Brooke be supporting equine welfare by generating broader welfare understanding in communities rather than just equine welfare? Would broader welfare development be more effective, or would it divert project time away from equids? - Can Brooke support the development of more affordable services so treatment options are more viable? Many countries work through private and government services, both of which require some financial contribution from the owners.   - Focus on generating viable services that are affordable? Create service providers with viable business models that will function independently of Brooke? |
| Unable to euthanase equids in extreme suffering. Leading to prolonged painful deaths and equine abandonment | - Cultural attitudes towards euthanasia – prohibit owners consenting - Availability of drugs or firearms to euthanase equids humanely - Availability of service providers with the skills/legal ability to euthanase equids safely - There is no economic benefit for an owner or a private service provider to euthanase an animal. An owner has to pay for drugs that will not get the equid better, the service provider misses out on income from treatments. | - Will Brooke ever be able to develop tertiary referral services (such as euthanasia) through local service providers. If not, what happens to these animals. Should Brooke continue to provide the services that cannot be provided locally? - if so Brooke will not be able to exit an area. If Brooke continues to provide these services will this stifle other service providers who may in the future be able to provide such services. - Support service providers to source affordable drugs/equipment for euthanasia, develop suppliers, subsidise so affordable to owners/users - Compensations: for euthanasia. Although this does increase euthanasia it can be abused, and does not necessarily encourage owners to euthanase their euqids for the right reasons. (although does this matter) - Advocacy working with governments to provide euthanasia services – as part of public health/food security. (roaming equines a disease risk) - Clarity needed about what is more important for the organisation developing sustainable services or alleviating immediate suffering – to support decision making in these situations. |
| Areas with a high turnover of animals.  People actively looking for equids in poor welfare, as cheap and they can work them and then abandon/sell. | - Difficult to achieve sustainable welfare improvement as new animals and/or owners each year - No long term economic benefits for keeping the animal healthy | - Can Brooke build wider empathy for equid welfare? Is it possible to build/train/develop welfare when only discussing one animal? Welfare as a concept applies to all animals. Should Brooke be supporting equine welfare by generating broader welfare understanding in communities rather than just equine welfare? Would broader welfare development be more effective, or would it divert project time away from equids? - Can Brooke support the development of more affordable services so treatment options are more viable? Many countries work through private and government services, both of which require some financial contribution from the owners. - Focus on generating viable services that are affordable? Create service providers with viable business models that will function independently of Brooke. Look for other actors who are supplying these equids in such poor condition – is this the better target? |
| Training private service providers who have no interest in welfare and only treat equids to make a profit:   - Irresponsible use of vet drugs - Not interested in treating equids as make more money from other animals | - Lack of empathy of service providers - Lack of demand for good services form owners. Owners don’t care or owners don’t know what a good service provision looks like - Privatised service provision model if unregulated can compromise welfare as profit put above animal needs (aspects such as euthanasia/pain relief not prioritised). This is a not unique to developing countries; in industrialised countries this is controlled through education, demand, regulation, legislation etc. | - Work with Local Service Providers and owners to build empathy, and demand for service provision quality - Work with advocacy actors to improve service provision infrastructure and regulation so welfare is prioritised (e.g. as done in developed world)   - Training institutions, veterinary boards and organisations – building professional integrity   - Support country to develop a regulatory system and infrastructure for professional development/training - Welfare training and social/welfare responsibility engrained in community using diverse approaches:   - Youth groups   - Schools   - Women’s groups   - Other organisations |
| Owner’s may not want to improve an equid’s welfare as this makes them harder to manage, handle or a target for theft.   - Male donkeys in Qualander communities India | - Owner lack motivation for change as brings no benefit - Unintended consequences of Brooke’s work, not bringing anticipated welfare changes – increased work, racing. | - Discuss with international teams how much of a problem is this? - Unintended consequences of the work. How will Brooke recognise this situation? |
| Peri-urban/urban environments. People won’t cooperate even if benefits are mutual. Community groups not available, more difficult to structure which limits the opportunity for some of Brooke’s current approaches | - Teams unclear about what motivates behaviours and therefore interventions are not appropriate or effective? - Sense of ‘community’ does not appear to exist, very difficult to achieve community engagement or social pressure, group formation not possible (which Brooke’s current programme model relies on.) | - Find different entry points. - Make use of other organisations links, groups and expertise. Brooke does not have to reinvent the wheel. (to do this we need to get other organisations interested in Brooke’s work – which may involve talking more widely about issues outside of equine welfare – livelihoods, women, animal healthcare rather than equid healthcare). |
| Working with migratory communities   - Brooke cannot work consistently with communities limiting the effectiveness of interventions |  | - Decide not to work with migratory communities and focus funding elsewhere. What happens to the equids? Is this ethical? Will this undermine Brooke’s reputation as migratory communities can be closely associated with permanent communities with which Brooke works? - Develop community influencers who spread the word while the communities are out of Brooke’s operational areas - Technology to provide distance support whilst in different areas. |
| **Organisational Questions that Arise from these No/Hard Win Situations** | | |
| Brooke does not currently have the capabilities to deal with these challenging behaviour change interventions. | - Teams do not have experience or experts in human behaviour change within teams. Therefore, teams are not able to analyse and identify root causes so interventions do not address the root causes of behaviour and are therefore not effective. - Teams are under pressure to covert spend to numbers (coverage and uptake) so interventions are superficial aimed at achieving maximum numbers for minimum spend meaning programmes themselves are not able to prioritise these more challenging scenarios | - If Brooke does not have the capabilities for behaviour change should it be focus on interventions that are more suited to current capabilities, or develop additional expertise to be more effective. - Brooke as an organisation needs very diverse skills set including welfare, veterinary, human behaviour change/development – how can the organisation obtain such skills in country and make the most of such varied capabilities. - Developing this wide skill based becomes increasingly challenging with decentralisation as people with these skills (particularly welfare/veterinary) are harder to recruit and support from the UK becomes more distant. - How can the UK better support the development of these skills in country. A large part of UK support focuses on technical training. Is this enough or are there other reasons why programmes are struggling to develop the necessary skills and how can these be addressed?) A lot of time is spent supporting programmes with technical information, do programmes need additional support with programme/intervention planning, strategic positioning, and management. - Some interventions are unsuccessful because they tackle the wrong behaviour or don’t address key barriers to behaviour. E.g. if owners are not motivated to do a new behaviour no matter how much you train them they will not change. - Brooke's current culture and ethos does not support outcome orientated work as it currently focuses on activities, outputs, coverage and uptake. This does not motivate teams to tackle these hard win situations as providing activities are still being done and coverage/uptake reported they go unrecognised. Changing this involves a significant culture shift with the organisation and different questions need to start asked of programmes to focus their project around outcome and impact rather than activities. |
| Ability to impact on welfare if Brooke’s argument is entirely economic | - There are many welfare improvements that can be made that will not bring an economic benefit, but are still critical to improving quality of life for equine animals. - Or the economic argument does enough to improve welfare sufficiently and additional investment to build empathy is not cost effective and often impossible. | - Analyse situation carefully and do full root cause analysis. Don’t make assumptions about why people act the way they do – ask them. - This assumes that communities are only driven by economic benefit and this has been shown not to always been the case. (e.g. social benefit) there are many examples equids playing an important social role (marriages etc.) - If Brooke truly wants to improve welfare sustainably then some degree of empathy is needed. Is economics enough for sustainable improvement or is an entry point to create interest and initial motivation. |
| Brooke is a charity, which suggests ‘giving’. | - Brooke is not clear with external audiences about what we ‘give’ as a charity. Is this a risk in the future - if programmes are increasingly building capacity rather than giving free equipment/services? | - Is this a problem? What do other charities do about this? |
| Conflicting messages and approaches with other charities or voluntary groups may undermine Brooke work | - Charities that provide free services and equipment can undermine Brooke’s capacity building and empowering approaches. - Credibility is risked with poor coordination or conflict between organisations. This has negative implications for future advocacy fundraising and Brooke’s reputation. | - Can in-country work be better coordinated by different charities? How can this coordination be achieved – centrally through head offices or through in country discussions? - Should Brooke be funding others rather than working in the same area? |
