# Supplementary material for: Improving working equine welfare in ‘hard-win’ situations, where gains are difficult, expensive or marginal
Source: PLoS One. 2018 Feb 6;13(2):e0191950. doi: 10.1371/journal.pone.0191950 (PMC5800664; doi:10.1371/journal.pone.0191950)
Supplement: S8 File — (DOCX) [file pone.0191950.s009.docx]

**Annex 2:** **Option and opportunities to tackle hard win situations better in the next strategic period**

| **Suggestion options for no/hard win situations** | **Rational and Benefit** | **Risks/ challenges** |
| --- | --- | --- |
| ‘Ring fenced funding for no/win hard win situations. | Limited and defined resources/time could be allocated to no/hard win areas, communities or work types. This would achieve short term welfare improvement/alleviation but not long term sustained changes. This would channel Brooke resources to work that yields the greater welfare improvement; improving programme efficiency. | No/hard win situations are not always clearly defined in isolated areas so ring fenced funding can be logistically impossible.  Is investing limited resources situation knowing there is going to be no long term benefit an effective or a good use of resources programme design?  If it is not possible to identify hard/no win situations before working in an area local stakeholders and team expectations be raised and then not met. |
| Focus on creating an enabling environment through advocacy | As explained no/hard win situations can be the results of deeper seated issues within the country, or communities. Brooke cannot always resolve such challenges at a local level and this may not be appropriate. National and intergovernmental advocacy offers an alternative opportunity to address such issues. It was consistently felt that advocacy work offered a critical component to Brooke’s programme model and a potential opportunity in no/hard win situations. | Impact from Advocacy work can be slow and implementation of changes at field level difficult. Marginalised communities may not benefit from advocacy driven changes. |
| Identifying other relevant actors to work with including partner organisations with different skill sets to tackle broader issues which impact on equine welfare. | Can Brooke re-think its work with partnerships to make better use of the complimentary skills other development organisation might have. This maybe more relevant for situations where Brooke is struggling to work due to deep rooted social issues. If these social issues can be linked to welfare problems then perhaps this is a potential partnership opportunity. (e.g. domestic violence linked with beating equids). | Working on social issues may results in mission creep, distracting teams away from animal welfare. This may be considered outside of mission scope and significant resources would be diverted away from welfare. Credible partner organisations may not be interested in working with Brooke because they cannot see how equine welfare links with their mission. For Brooke to work with other credible organisations it may have to make more visible links to development, and recognise that this could involve discussion issues that are outside of the mission. |
| Could Brooke make use of other actors when working with owners and the usual service providers is not effective | Are there other actors who could have a bigger indirect influence on equid welfare when working with owners and users is not effective:   - Youth in communities – especially if trying to achieve the next generation change - Employers of equine owners who control the working environment – e.g. brick kilns owners - Women | Programmes need to ensure they target the most relevant ‘actor(s)’. This relate back to identifying the root causes of the current behaviours and the barriers to new behaviours and understanding who can influence this most. |
| Recognising no Win situations early and accepting that the Brooke cannot be effective everywhere | Can particularly challenging hard win situations be identified through scoping – so they can be identified and tackled proactively rather than reactively? Given that so many no/hard wins are involve challenging behaviour change scenarios, can behaviour drivers/barriers be assessed during scoping to give insight into ease of work? Measuring behaviour drivers/barriers is complex and likely to involve proxy indicators, but Brooke could consult expertise for this support.  Programmes need to be measuring change (Outcomes/impact), not just activities, to better understand the change being made. If Brooke can strengthen and prioritise outcome/impact evaluation within programmes Hard win situations will be identified earlier, so can either be tackled or exited. | It is very difficult and expensive to get reliable information during scoping and it may be too ambitious/expensive to collect this level of detail.  Behaviour drivers may only be evident once Brooke has started working in an area.  Outcome/impact monitoring is difficult and costly (time and money) so will be a significant investment for the organisation. The organisation will only value this monitoring if the results are used as a basis for decision making (e.g. funding, expansion etc)  How does Brooke measure change in constantly changing populations of equids? In these populations overall welfare improvement is diluted out by a constant influx of ‘new’ equids in poor welfare. |
| Accepting there may be some issues, communities or places where Brooke cannot effectively work | Can Brooke ever work effectively in extremely poor communities who lack fundamental human rights? Is it ethical to have projects promoting equid welfare is such communities? Will communities that have a very high time preference (make decisions based on the immediate rather than the longterm) ever recognise the value/economic benefit of improving welfare? i.e. the “time preference” concept used in health economics | Improving equine welfare may improve their essential income, so even if owners’ struggle to see the long term benefit, it is in their interest to support them to achieve this. |
| Taking difficult decision more quickly when no win/Hard win Situations arise | When no/hard win situations are identified programmatic action needs to be taken without unnecessary delay. Decision making responsibility must be clear and it must be supported by transparent guidance on how to take decisions. Decision must be based on relevant /reliable evidence and has to be taken in the light of each context. | Decision making is difficult and it is hard to equip teams with the skills necessary. Decisions are complicated by external influencers, team loyalties, reputational risk of leaving areas and internal and external politics. |
| Strengthen Brooke’s capability to develop truly community centred interventions to trigger lasting and relevant behaviour change in key actors | Many no/hard win situations involved barriers to human behaviour change so perhaps this is an area where Brooke needs additional capability. Brooke has welfare and veterinary at its core, however human behaviour change is science in itself and perhaps teams need more technical expertise in this subject.  Is it possible for Brooke to convert new behaviours in habit – to guarantee that they are continued even after exit? | It is very difficult to find teams with human behaviour change/community based approaches experience alongside the technical welfare/veterinary knowledge needed to drive the organisation’s core mission.  If the organisation becomes too focused on human behaviour changes there is a risk that animal welfare may get lost.  Creating habits (unconscious behaviour) is extremely challenging and not always possible. |
| Decentralised decision making so teams have more flexibility in the field, so activities can be tailored to context, even to community. | A huge proportion of organisational decision making is escalated vertically, both in country and in the UK. This can paralyse field and support teams, stifling innovation. This is coupled with a fear of ‘messing up’ leading to huge risk aversion. If decision making can be effectively delegated this may make field teams more flexible and innovative. | Decentralisation is difficult given the high level of technical capacity (welfare and veterinary) needed for Brooke’s work. This technical expertise is not available in almost all the countries in which Brooke works so support is needed and this limits and challenges the degree of decentralisation.  Decentralisation has been tried and tested by other development organisation and the Brooke can learn from these experiences, to avoid reinventing the wheel. |
| Shifting organisational focus to magnitude of impact rather than number of activities | If Brooke wants teams to focus on quality of impact this has to be prioritised above activity reporting. This may only be achieved if Brooke is willing to reduce the degree of output reporting in the organisation so programmes have the time space and incentive to focus on impact. | For Brooke to reduce activity reporting it must have a reliable way to measure outcome and impact to ensure the work is effective and accountable. This will take time and resources to develop. |
